# Loss of aPKCλ in Differentiated Neurons Disrupts the Polarity Complex but Does Not Induce Obvious Neuronal Loss or Disorientation in Mouse Brains

**DOI:** 10.1371/journal.pone.0084036

**Published:** 2013-12-31

**Authors:** Tomoyuki Yamanaka, Asako Tosaki, Masaru Kurosawa, Kazunori Akimoto, Tomonori Hirose, Shigeo Ohno, Nobutaka Hattori, Nobuyuki Nukina

**Affiliations:** 1 Laboratory for Structural Neuropathology, RIKEN Brain Science Institute, Saitama, Japan; 2 Department of Neuroscience for Neurodegenerative Disorders, Juntendo University Graduate School of Medicine, Tokyo, Japan; 3 Department of Molecular Medical Science, Faculty of Pharmaceutical Sciences, Tokyo University of Science, Chiba, Japan; 4 Department of Molecular Biology, Yokohama City University Graduate School of Medical Science, Yokohama, Japan; 5 Department of Neurology, Juntendo University Graduate School of Medicine, Tokyo, Japan; 6 Core Research for Evolutionary Science and Technology, Japan Science and Technology Agency, Tokyo, Japan; SUNY Downstate Medical Center, United States of America

## Abstract

Cell polarity plays a critical role in neuronal differentiation during development of the central nervous system (CNS). Recent studies have established the significance of atypical protein kinase C (aPKC) and its interacting partners, which include PAR-3, PAR-6 and Lgl, in regulating cell polarization during neuronal differentiation. However, their roles in neuronal maintenance after CNS development remain unclear. Here we performed conditional deletion of aPKCλ, a major aPKC isoform in the brain, in differentiated neurons of mice by camk2a-cre or synapsinI-cre mediated gene targeting. We found significant reduction of aPKCλ and total aPKCs in the adult mouse brains. The aPKCλ deletion also reduced PAR-6β, possibly by its destabilization, whereas expression of other related proteins such as PAR-3 and Lgl-1 was unaffected. Biochemical analyses suggested that a significant fraction of aPKCλ formed a protein complex with PAR-6β and Lgl-1 in the brain lysates, which was disrupted by the aPKCλ deletion. Notably, the aPKCλ deletion mice did not show apparent cell loss/degeneration in the brain. In addition, neuronal orientation/distribution seemed to be unaffected. Thus, despite the polarity complex disruption, neuronal deletion of aPKCλ does not induce obvious cell loss or disorientation in mouse brains after cell differentiation.

## Introduction

In mammals, neuronal cells are polarized in multiple steps of cell differentiation. These include apical-basal polarity of neuronal progenitor epithelial cells, asymmetric division of the progenitors, directed cell migration, axon-dendrite specification and dendritic spine formation. These cell polarizations are fundamental to proper development of the central nervous system (CNS).

Atypical protein kinase C (aPKC) is a Ser/Thr kinase that is structurally different from other typical PKC subfamily kinases; that is, it lacks binding regions for calcium and phorbol ester in its regulatory domain, but contains a protein binding PB1 domain at its N-terminus [1]. aPKC forms an evolutionarily conserved protein complex with the PDZ-containing proteins PAR-3 and PAR-6, and it localizes asymmetrically within a cell to regulate polarization. This has been observed in various types of cells, such as *C. elegans* one-cell embryos, *Drosophila* epidermis and mammalian epithelial cells [2]–[4]. aPKC also forms a complex with Lgl, a protein that contains WD repeats. This complex forms independently of PAR-3 and regulates aPKC/PAR-3/PAR-6-mediated polarization of epithelial cells [5]–[8]. Recent studies of gene knockout or knockdown in mice have established the *in vivo* significance of aPKCλ and PAR-3 for epithelial tissue morphogenesis and its maintenance in mammals [9]–[14].

Genetic studies using *Drosophila* have further identified critical roles of aPKC/PAR-3/PAR-6 and Lgl in CNS development through the regulation of asymmetric division of neuronal progenitors (neuroblasts) [15]–[17]. Previously, we found that conditional knockout of an aPKC isoform—aPKCλ—in mice using a nestin-cre transgene induces disruption of apical-basal polarity of neuronal progenitor cells (neuroepithelial cells) in mouse brain cortex [18]. Although the role of aPKCλ in neuronal progenitor differentiation was not clarified by this study, possibly because gene knockout was done at a relatively late stage (E15), knockdown of PAR-3 at earlier stages (E12∼13) enhances neuronal progenitor differentiation whereas ectopic expression of PAR-3 or PAR-6 suppresses it in mouse brains [19], [20]. In contrast, knockout of the Lgl isoform Lgl-1 suppresses progenitor differentiation and induces its continuous proliferation, leading to neoplasia formation [21], suggesting that neuronal progenitor differentiation is differentially regulated by PAR-3 and Lgl-1 in mammals. The importance of aPKC for neural progenitor proliferation/differentiation is shown during neurogenesis in Xenopus [22], [23] and zebrafish [24] embryos. As for neuronal migration, overexpression of the PAR-6 isoform PAR-6α has been shown to suppress migration of cerebellar granule neurons by disturbing cytoskeletal organization [25], [26]. Thus, aPKC and/or its interactors are involved in multiple steps of CNS development from progenitor maintenance/differentiation to cell migration by regulating cell polarization.

Studies using *in vitro* cultured rat hippocampal neurons further suggest the involvement of aPKC/PAR-3/PAR-6 in later stages of differentiation [27], [28]. One of them is axon specification, during which these proteins localize to the tip of the growing axon and regulate axonal growth by interacting with several molecules such as KIF3A, APC and Tiam1 [29]–[32]. In addition, TGF-β signaling and Smurf1 E3 ligase regulate PAR-6 by its phosphorylation and degradation, respectively, and play a role in axonal growth of cortical neurons during mouse brain development [33], [34]. Lgl-1 has also been shown to regulate axonal growth of rat cortical neurons *in vivo* [35]. PAR-3, aPKC and PAR-6 are required for dendritic spine morphogenesis in *in-vitro* cultured hippocampal neurons [36], [37], and the potential *in vivo* significance of this is suggested by evidence that BAI1 interacts with PAR-3 to recruit it to dendritic spines in mice [38]. In addition, analysis of mutant zebrafish has revealed that aPKCλ is required for dendritic specification of Purkinje cells during development [39]. Thus, although these observations contradict those observed in *Drosophila* [40], at least in mammals (and possibly also in zebrafish), aPKC and its interactors are involved in axon/dendrite specification and morphogenesis in later stages of neuronal differentiation.

In contrast to the significance of aPKC and its interactors for neuronal differentiation during CNS development, their roles in neuronal maintenance after CNS development remain unknown. To clarify this, we established mice in which aPKCλ is deleted specifically in differentiated neurons. We found a significant reduction of aPKCλ and the polarity complex in the brains of these mice. However, the mice were healthy and did not show clear brain weight loss or cell degeneration. In addition, staining of several markers suggested that neuronal orientation/distribution was totally unaffected in these mice. Thus, despite the disruption of the polarity complex, our analysis did not detect obvious cell loss or disorientation by neuronal deletion of aPKCλ after cell differentiation.

## Results

### Promoter- and age-dependent DNA recombination in brain neurons by cre transgenes

To examine the role of aPKCλ in differentiated mouse neurons, we used a cre-loxP system to establish mouse lines with conditional deletion of aPKCλ in differentiated neurons [41]. For cre expression, we used two transgenic mouse lines, synapsinI-cre (S1-cre) and camk2a-cre (C2-cre), which express cre specifically in differentiated, postmitotic neurons of the brain [41], [42]. We first checked cre expression by these transgenes using RNZ reporter mice that express LacZ in nuclei by cre-mediated DNA recombination [43]. LacZ staining using X-gal as substrate revealed that S1-cre induced LacZ expression in whole brain regions, especially in layers IV/V of cortex, CA3 and dentate gyrus of the hippocampus, thalamus and brain stem (Figure S1A, B). In contrast, C2-cre induced LacZ expression specifically in the forebrain, especially in layers II-IV of cortex and CA1/3 and dentate gyrus of the hippocampus (Figure S1C, D).

**Figure 1:**
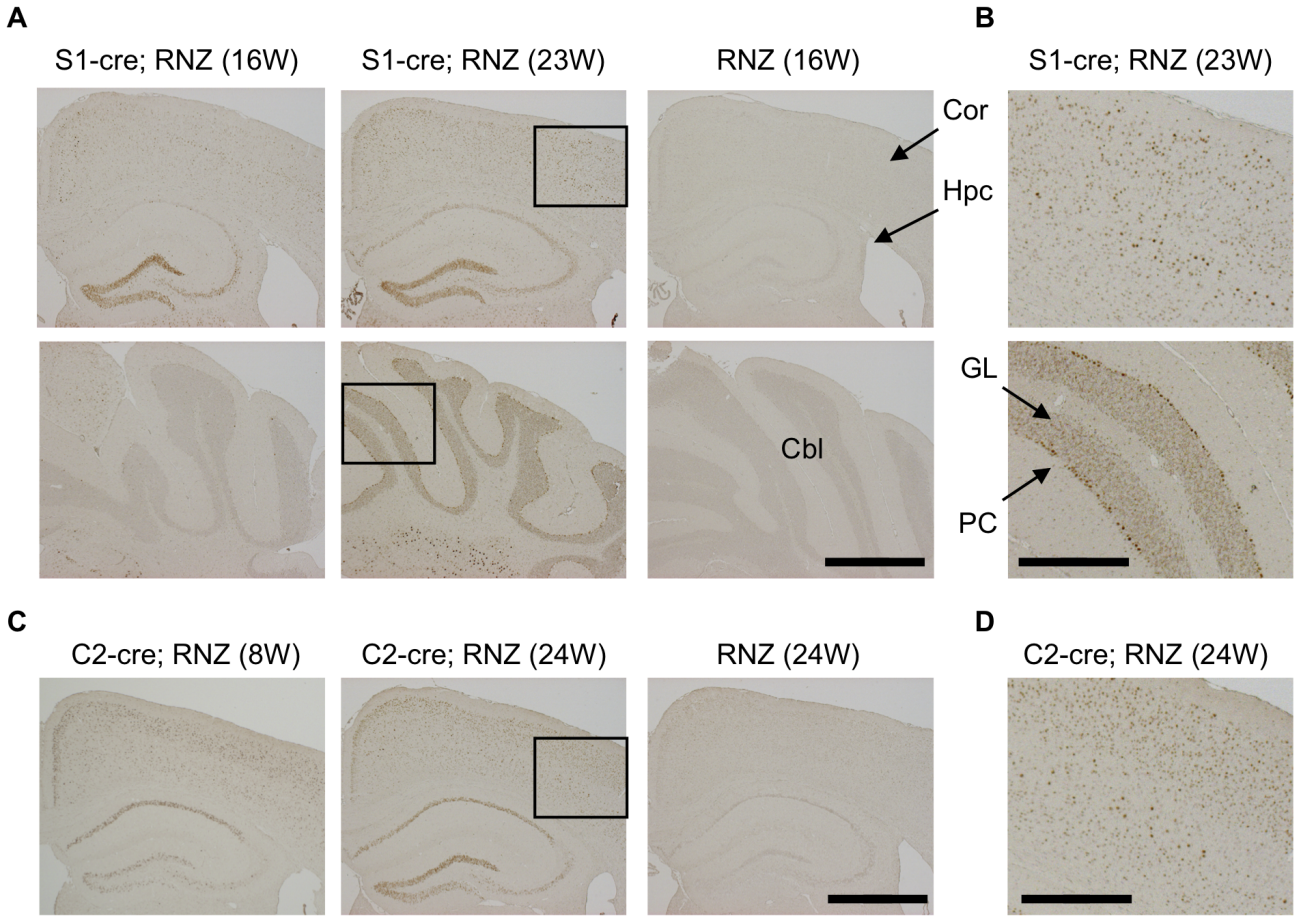
Detection of DNA recombination by synapsinI-cre or camk2a-cre transgene in mouse brain. Transgenic mice for synapsinI-cre (S1-cre) or camk2a-cre (C2-cre) were crossed with RNZ mice. RNZ male mice harboring S1-cre or C2-cre at indicated weeks of age were subjected to anti-LacZ staining to detect cre-mediated DNA recombination. RNZ mice without a cre transgene were used as controls. (A) In S1-cre; RNZ mice at 16 weeks of age, LacZ-positive cells were strongly detected in dentate gyrus and CA3 in hippocampus and some cortical cells, but very few were seen in cerebellum. At 23 weeks, LacZ expression became wider in the cortex and hippocampus, and was clearly detected in cerebellum. No distinct LacZ expression was detected in the control RNZ mice. (B) High magnification of boxed region in (A). LacZ expression was broadly detected in multiple layers of cortex and Purkinje and granular cells of cerebellum in 23 week-old S1-cre; RNZ mice. (C) LacZ-positive cells were broadly detected in brains of 8-week-old C2-cre; RNZ mice, especially in layer II/III of cortex and CA1 of hippocampus. It became wider at 24 weeks of age. Again, no distinct LacZ expression was observed in the control RNZ mice. (D) High magnification of boxed region in (C), indicating detection of LacZ-positive cells in multiple layers of cortex in 24 week-old C2-cre; RNZ mice. Cor (cortex), Hpc (hippocampus), Cbl (cerebellum), PC (Purkinje cell) and GL (granular layer). Bars are 1 mm (A, C) and 0.4 mm (B, D).

We also checked cre-mediated LacZ expression by staining with anti-LacZ antibody. In S1-cre; RNZ mice, LacZ-positive cells were strongly detected in the hippocampus and cortex but very few were seen in the cerebellum at 16 weeks (Figure 1A), consistent with the above LacZ staining data. The specificity of these signals was confirmed using RNZ mice without the cre transgene in which distinct anti-LacZ signal was not detected (Figure 1A). Notably, the LacZ expression became wider at a later stage (23 weeks): anti-LacZ positive cells were more broadly detected in cortex and hippocampus (Figure 1A, B). Especially in cerebellum, significant anti-LacZ signals were detected in Purkinje and granular cells at 23 weeks this stage (Figure 1A, B). Similarly, cells with high LacZ expression were more broadly detected in cortex and hippocampus of C2-cre; RNZ mice at 24 weeks of age compared with those at 8 weeks of age, although the expression was restricted to the forebrain region in these mice (Figure 1C, D). Thus, cre expression in S1-cre or C2-cre is promoter dependent and become wider with age in mouse brain.

### Generation of mutant mice with conditional aPKCλ deletion in differentiated neurons

The cre transgenic mice were crossed with aPKCλ flox mice in which exon 5 of aPKCλ genes is flanked by *loxP* sequences [18]. To generate aPKCλ conditional deletion mice under the C2-cre transgene (aPKCλ C2-cko), we crossed aPKCλ flox/+; C2-cre mice with aPKCλ flox/flox mice. Resultant pups were aPKCλ C2-cko (flox/flox; C2-cre) mice at the expected Mendelian ratio in addition to mice with other genotypes (Table S1). The aPKCλ conditional deletion mice under the S1-cre transgene (aPKCλ S1-cko) were generated by a similar strategy. In this case, however, we occasionally obtained mice with a deleted aPKCλ allele, possibly due to its recombination in the germline during generation [44]. As a consequence, two types of aPKCλ S1-cko mice were obtained; flox/flox; S1-cre and flox/−; S1-cre (Table S2), although the ratio for these cko mice was a little higher than expected, for an unknown reason. Thus, we obtained two lines of differentiated neuron-specific aPKCλ conditional deletion mice, aPKCλ S1-cko and aPKCλ C2-cko mice.

### Neuronal deletion of aPKCλ results in reduction of total aPKCs and PAR-6β in mouse brain

To check the expression of aPKCλ and its related proteins in aPKCλ S1-cko and aPKCλ C2-cko mice, we first performed Western blot analysis. Because of age-dependent cre expression in the mouse brain as described above, we sampled brains at later stages (7-month-old aPKCλ S1-cko mouse and 13-month-old aPKCλ C2-cko mouse) to delete aPKCλ in broad types of cells in the brain. The brains were then separated into 5 regions: striatum (Str), hippocampus (Hpc), cortex (Cor), other remaining cerebrum regions (Other) and cerebellum (Cbl), and analyzed by Western blotting.

**Figure 2:**
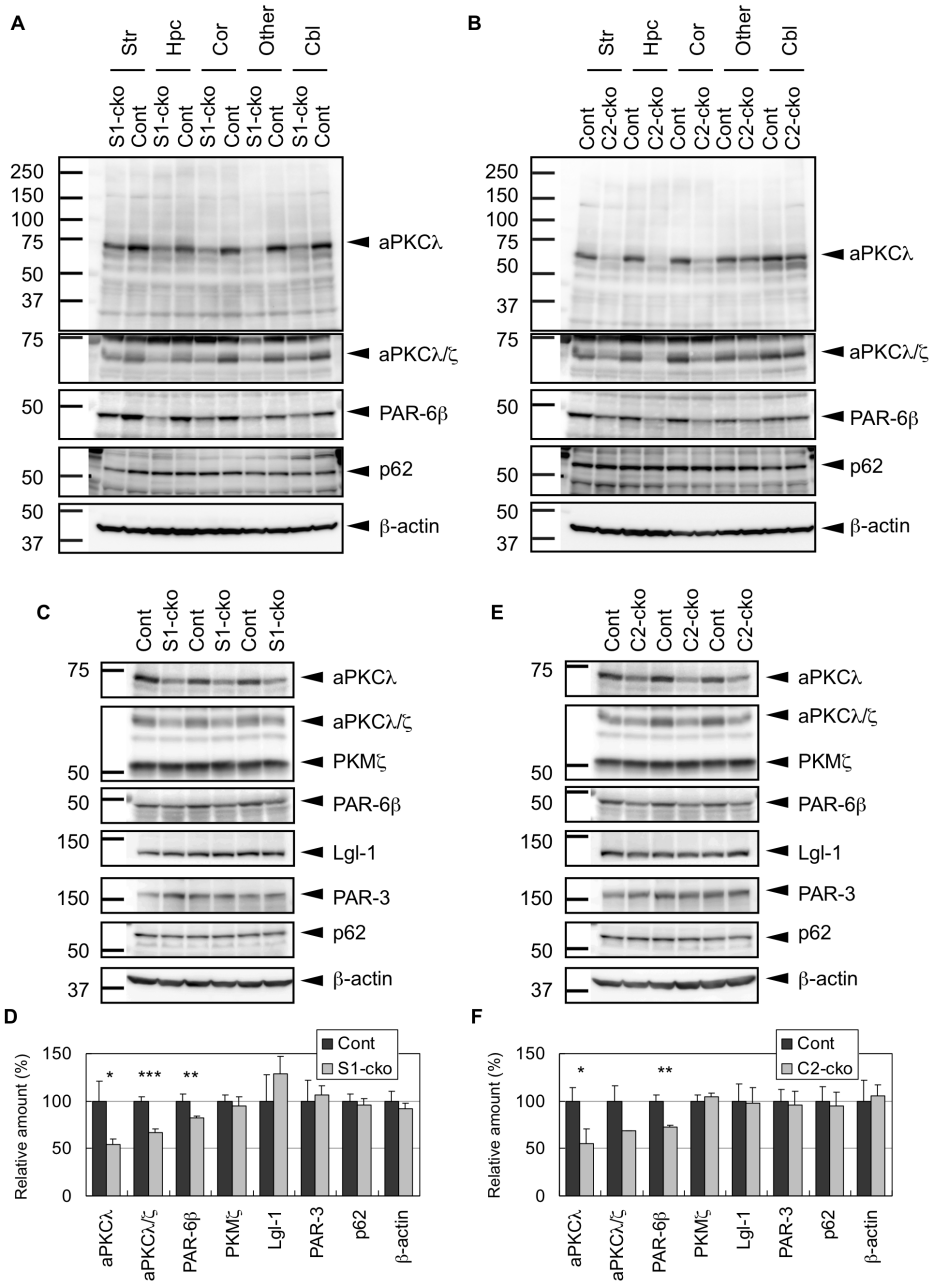
Western blot analysis of aPKCλ and its interacting proteins in the brain of aPKCλ deletion mice. (A) Brains of 7-month-old female mice harboring aPKCλ flox/−; S1-cre (S1-cko) or flox/+ (Cont) were separated into five regions: striatum (Str), hippocampus (Hpc), cortex (Cor), other remaining cerebrum regions (Other) and cerebellum (Cbl). These tissue regions were subjected to Western blot analysis using antibody specific to aPKCλ (BD, 610175) or antibody recognizing both aPKCλ/ζ (Santa Cruz (SC), sc-216). Antibodies for PAR-6β, p62 and β-actin were also used for the analysis. (B) Brains of 13-month-old female mice harboring aPKCλ flox/+ (Cont) or flox/flox; C2-cre (C2-cko) were separated and analyzed as in (A). (C) Total cerebrum of 11-month-old female mice harboring aPKCλ flox/flox; S1-cre (S1-cko; n = 3) or flox/flox (Cont; n = 3) were subjected to Western blot analysis using anti-aPKCλ (BD) or anti-aPKCλ/ζ (SC). An alternative isoform of aPKCζ, PKMζ was also detected by anti-aPKCλ/ζ (SC). Antibodies for PAR-6β, PAR-3, Lgl-1, p62 and β-actin were also used for the analysis. (D) Bands in (C) were quantified and plotted. (E) Total cerebrum of 20-month-old female mice harboring aPKCλ flox/flox; C2-cre (C2-cko; n = 3) or flox/flox (Cont; n = 3) were subjected to Western blot analysis as in (C). (F) Bands in (E) were quantified and plotted. Values are means ±SD (*P<0.05, **P<0.01, ***P<0.001).

Staining with aPKCλ-specific antibody revealed that aPKCλ expression, which was widely detected in the brain, was reduced in aPKCλ S1-cko mouse brain (Figure 2A). Similar reduction was also observed when we used an antibody recognizing both aPKCλ and another aPKC isoform, aPKCζ [18] (Figure 2A), suggesting that expression of all aPKC isoforms was reduced in these brain regions of aPKCλ S1-cko mice. In addition to aPKCs, we found that expression of PAR-6β, a PAR-6 isoform that binds to the PB1 domain of aPKCλ to form a functional protein complex for cell polarization [3], [4], [45], was also reduced in aPKCλ S1-cko mouse brains (Figure 2A). In contrast, p62, another PB1-interacting protein of aPKCλ [46], [47], did not show altered expression after aPKCλ deletion (Figure 2A). Similar patterns of altered aPKCλ, total aPKCs and PAR-6β expression, but not p62, were observed in aPKCλ C2-cko mice, although the alterations were specific to forebrain regions including striatum, hippocampus and cortex (Figure 2B), consistent with the C2-cre expression described above (Figure 1, S1). These data support the region-dependent deletion of aPKCλ by the S1-cre or C2-cre transgene, which accompanies reductions of total aPKCs and PAR-6β in the brain.

The reduction of aPKCλ, total aPKCs, and PAR-6β was also observed when we used cerebra of aPKCλ S1-cko (Figure 2C, D) or C2-cko (Figure 2E, F) mice. In contrast, expression of other aPKCλ interacting polarity proteins, such as PAR-3 and Lgl-1, as well as p62, was not altered (Figure 2C–F). In addition, PKMζ, an alternative isoform of aPKCζ lacking its N-terminal regulatory domain [48], did not show altered expression in aPKCλ deletion cerebra (Figure 2C–F). Thus, aPKCλ deletion by S1-cre or C2-cre induces specific reduction of aPKCλ, total aPKCs and PAR-6β without affecting expressions of PAR-3, Lgl-1, p62 and PKMζ in the cerebrum. Taken together, these data support the notion of aPKCλ gene knockout by cre transgenes, which results in ∼50% reduction of total aPKCs in the brain. The remaining aPKCs after aPKCλ conditional deletion might be expressed in non-neuronal cells such as glia and/or neurons without cre expression.

### aPKCλ is a major aPKC isoform in mouse brain and its deletion did not affect transcription of its related gene

**Figure 3:**
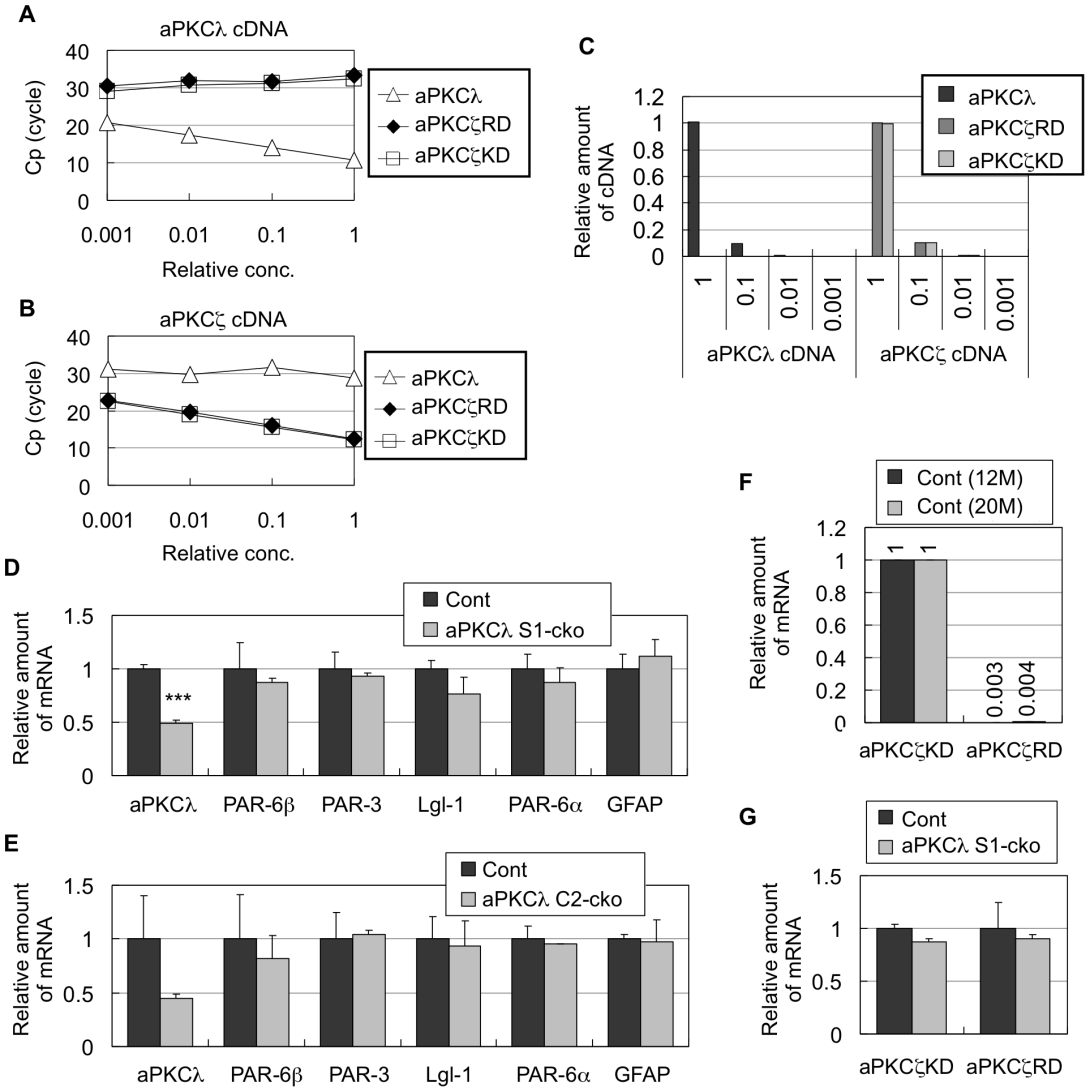
Quantitative RT-PCR of aPKCλ and its related genes in aPKCλ conditional deletion mice. (A, B) Specificity of primer sets for aPKCλ and aPKCζ for quantitative PCR. Plasmid DNA for mouse aPKCλ cDNA (A) or aPKCζ cDNA (B) at indicated relative concentrations was subjected to real-time PCR using primers of aPKCλ and aPKCζ (RD or KD). Crossing point (Cp) means the cycle number of first detection of positive signal for PCR product. Low Cp indicates efficient detection of template cDNA, whereas high Cp without inverse correlation with the amount of the input indicates no significant detection. (C) Data in (A) and (B) were used for quantification of relative amounts of cDNA. Specific amplifications by each primer set were confirmed. (D, E) Quantitative RT-PCR of aPKCλ and indicated genes in cerebra of 11-month-old male mice harboring aPKCλ flox/flox (Cont) or aPKCλ flox/flox; S1-cre (aPKCλ S1-cko) (D, n = 3 for each), or 20-month-old male mice harboring aPKCλ flox/flox (Cont) or aPKCλ flox/flox; C2-cre (aPKCλ C2-cko) (E, n = 3 for each). Amounts relative to control are shown. (F) Quantitative RT-PCR of aPKCζ in cerebra of aPKCλ flox/flox (Cont) male mice at 11 months or 20 months of age (n = 3 for each). aPKCζ plasmid DNA was used as standard for quantification. Primer sets for aPKCζ RD and KD were used to detect full-length aPKCζ and both aPKCζ/PKMζ, respectively. The values obtained by aPKCζ KD primer set were taken as 1. (G) Quantitative RT-PCR of aPKCζ and PKMζ in cerebra of 11-month-old male mice harboring aPKCλ flox/flox (Cont) or aPKCλ flox/flox; S1-cre (aPKCλ S1-cko) (n = 3 for each). Amount relative to control is shown. Values are means ±SD.

We next examined mRNA levels of aPKCλ and other related genes in the cerebrum of aPKCλ deletion mice by quantitative RT-PCR. First, we made a primer set for aPKCλ targeting its regulatory domain (RD) and two primer sets for aPKCζ targeting its RD and kinase domain (KD) (see Materials and Methods). To check the specificity of these primer sets, we used plasmid DNA containing mouse aPKCλ or aPKCζ cDNA for quantitative PCR. As shown in Figure 3A and B, the aPKCλ primer set efficiently amplified only aPKCλ cDNA, whereas aPKCζ primer sets (RD and KD) efficiently amplified only aPKCζ cDNA. Quantification confirmed specific detection of the target genes by these primer sets (Figure 3C). Thus, the primers we used are available for aPKC isoform-specific detection by quantitative PCR. RT-PCR using the aPKCλ primer set indicates around 50% reduction of aPKCλ in cerebrum of aPKCλ S1-cko or C2-cko mice (Figure 3D, E), which is compatible with the Western blot data (Figure 2D, F). In contrast, the aPKCλ deletion did not affect mRNA expressions of PAR-6β, PAR-3, Lgl-1 and PAR-6α, another PAR-6 isoform (Figure 3D, E). No reduction of PAR-6β mRNA in contrast to its protein reduction by aPKCλ deletion suggests that PAR-6β protein reduction is not caused by its reduced transcription, but rather by other unknown mechanisms such as destabilization.

Using the plasmid DNA of aPKCζ, we estimated the relative amount of aPKCζ (full-length) with that of PKMζ in mouse brain. The RD and KD primer sets for aPKCζ were used to detect aPKCζ and both aPKCζ/PKMζ, respectively. As shown in Figure 3F, PCR amplification by the aPKCζ RD primer set was hardly observed compared with that by the aPKCζ KD primer set. Thus, PKMζ is the abundant isoform in adult mouse brain, which is consistent with previous observations [18], [48], [49]. We also observed that expressions of aPKCζ and PKMζ were not altered in aPKCλ S1-cko brains (Figure 3G), suggesting no compensatory induction of aPKCζ/PKMζ by aPKCλ deletion. Taken together with the Western blot data described above (Figure 2), these data support the notion that aPKCλ is a major full-length aPKC isoform expressed in adult mouse brain and that aPKCλ deletion mostly reflects loss of total aPKCs in neurons.

### Neuronal deletion of aPKCλ disrupts polarity protein complex in mouse brain cortex

**Figure 4:**
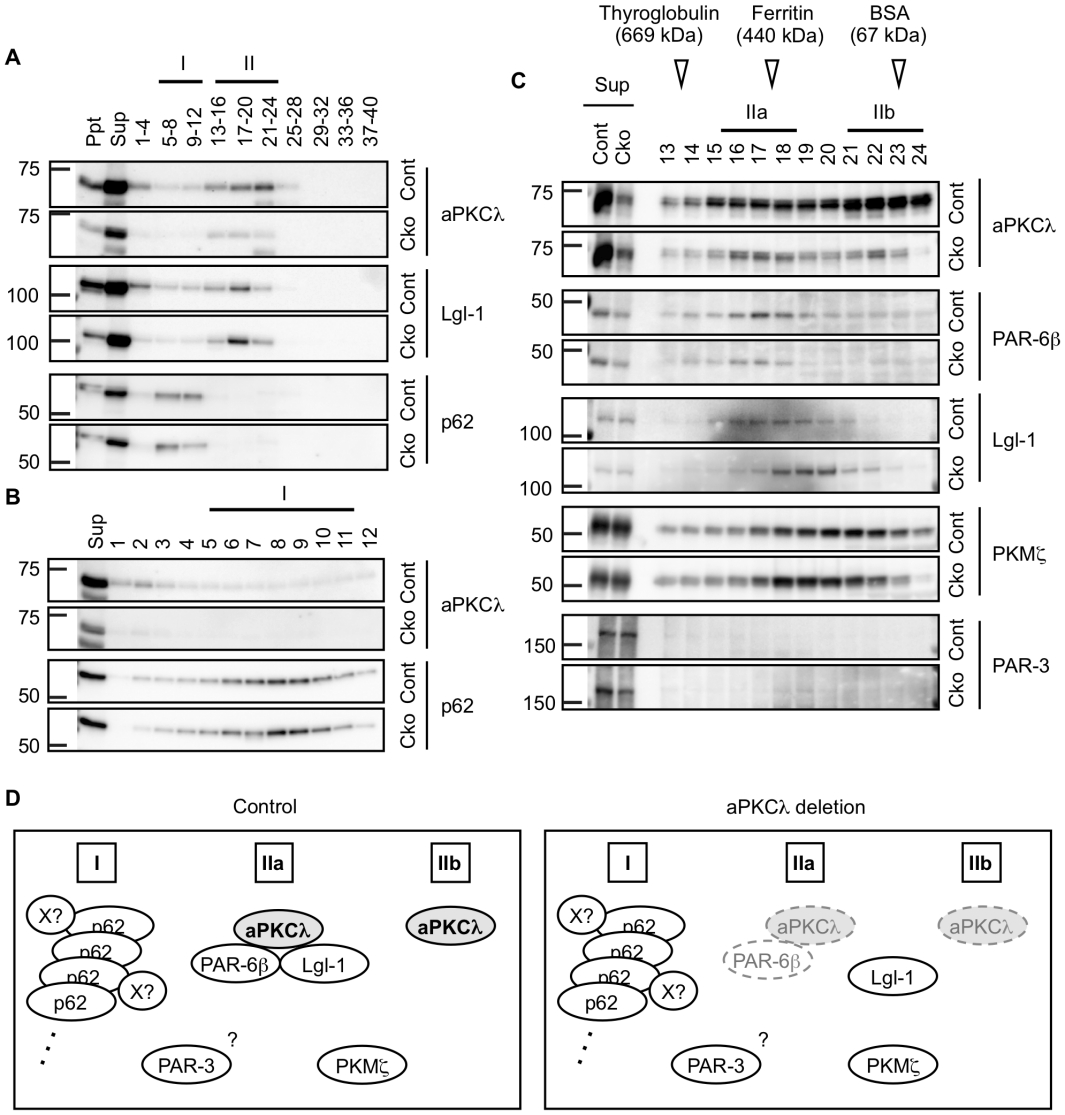
Gel filtration of cortical lysates of aPKCλ conditional deletion mice. Cortex of 7-month-old female mice harboring aPKCλ flox/−; S1-cre (Cko) or flox/+ (Cont) was homogenized with lysis buffer. After centrifugation and removal of pellets (Ppt), supernatants (Sup) were subjected to gel filtration, and a total of 40 fractions were collected. Molecular weight markers were detected in Fr. 13–14 (669 kDa; thyroglobulin), Fr. 17–18 (440 kDa; ferritin), Fr. 23 (67 kDa; bovine serum albumin) and Fr. 29 (25 kDa; RNase). (A) Western blot analysis of Ppt, Sup, and mixture of four sequential fractions using antibodies for aPKCλ, Lgl-1 and p62. aPKCλ and Lgl-1 were detected mainly in fractions 13–24 (referred to as Fr. II) in the control cortex, whereas p62 was detected exclusively in fractions 5–12 (Fr. I). (B) Western blot analysis of Sup and fractions 1–12 using antibodies for aPKCλ and p62. p62 but not aPKCλ was highly detected in the Fr. I in control cortex. (C) Western blot analysis of Sup and fractions 13–24 using antibodies for aPKCλ, PAR-6β, Lgl-1, PKMζ (sc-216) and PAR-3. aPKCλ was broadly detected in fractions 15-24 in the control cortex, which could be separated into two fractions; Fr. IIa containing PAR-6β and Lgl-1, and Fr. IIb without containing aPKCλ-interacting proteins examined here. (D) Schematic model of potential protein compositions in the cortical lysates. In the control mouse, aPKCλ was incorporated into two major fractions: the Fr. IIa containing aPKCλ in a protein complex with PAR-6β and Lgl-1, and the Fr. IIb containing complex-free aPKCλ monomer. In contrast, aPKCλ was not clearly detected in the Fr. I containing large protein complex composed of p62 oligomer and some of its interacting proteins (indicated by an X). aPKCλ deletion induces reductions of aPKCλ in complex (IIa) as well as free aPKCλ (IIb), resulting in PAR-6β reduction and Lgl-1 dissociation from the complex.

For regulation of cell polarity, aPKCλ works as a protein complex with other polarity proteins including PAR-6, Lgl-1 and PAR-3 [3], [4], [7]. To examine the effect of aPKCλ deletion on the protein complex formation, we lysed the cortex of aPKCλ S1-cko mice and subjected it to gel filtration. As shown in Figure 4A, most of the aPKCλ was solubilized in this condition. Gel filtration revealed that aPKCλ and Lgl-1 were mainly found in fractions 13–24 (referred to as Fr. II in Figure 4A). Reduction of aPKCλ in Fr. II was observed in aPKCλ S1-cko cortical lysates. In contrast, p62 was found in earlier fractions 5–12 (referred to as Fr. I in Figure 4A), suggesting that it incorporates into a large protein complex. Detailed analysis of fractions 1–12 suggests that p62 was mainly contained in Fr. I where aPKCλ was hardly detected (Figure 4B). Thus, aPKCλ and Lgl-1 were contained in Fr. II and segregated from Fr. I, which contained p62 in mouse cortical lysates. Detailed analysis of fractions 13–24 (Figure 4C) suggests that Fr. II was roughly segregated into two fractions: IIa containing aPKCλ and its interacting proteins PAR-6β and Lgl-1; and IIb containing only aPKCλ. PKMζ was detected broadly in fractions 13–24 and the peak fractions did not overlap with IIa and IIb, whereas PAR-3 was not clearly detected in these fractions. aPKCλ deletion resulted in reductions of aPKCλ (IIa and IIb) and PAR-6β (IIb), and a shift of Lgl-1 (IIb) to later fractions.

These gel filtration data are summarized as a hypothetical model in Figure 4D. We suggest that aPKCλ exists as two states: aPKCλ in the complex with PAR-6β and Lgl-1 in Fr. IIa; and aPKCλ as free monomer in Fr. IIb. PAR-3 and PKMζ may not significantly interact with aPKCλ in the lysates. In contrast, p62 exists as large complex possibly containing oligomers through its PB1-PB1 trans-interactions and some of its interactors in Fr. I. aPKCλ deletion induced reductions of aPKCλ and PAR-6β and dissociation of Lgl-1, resulting in disruption of the polarity protein complex in the cortex.

**Figure 5:**
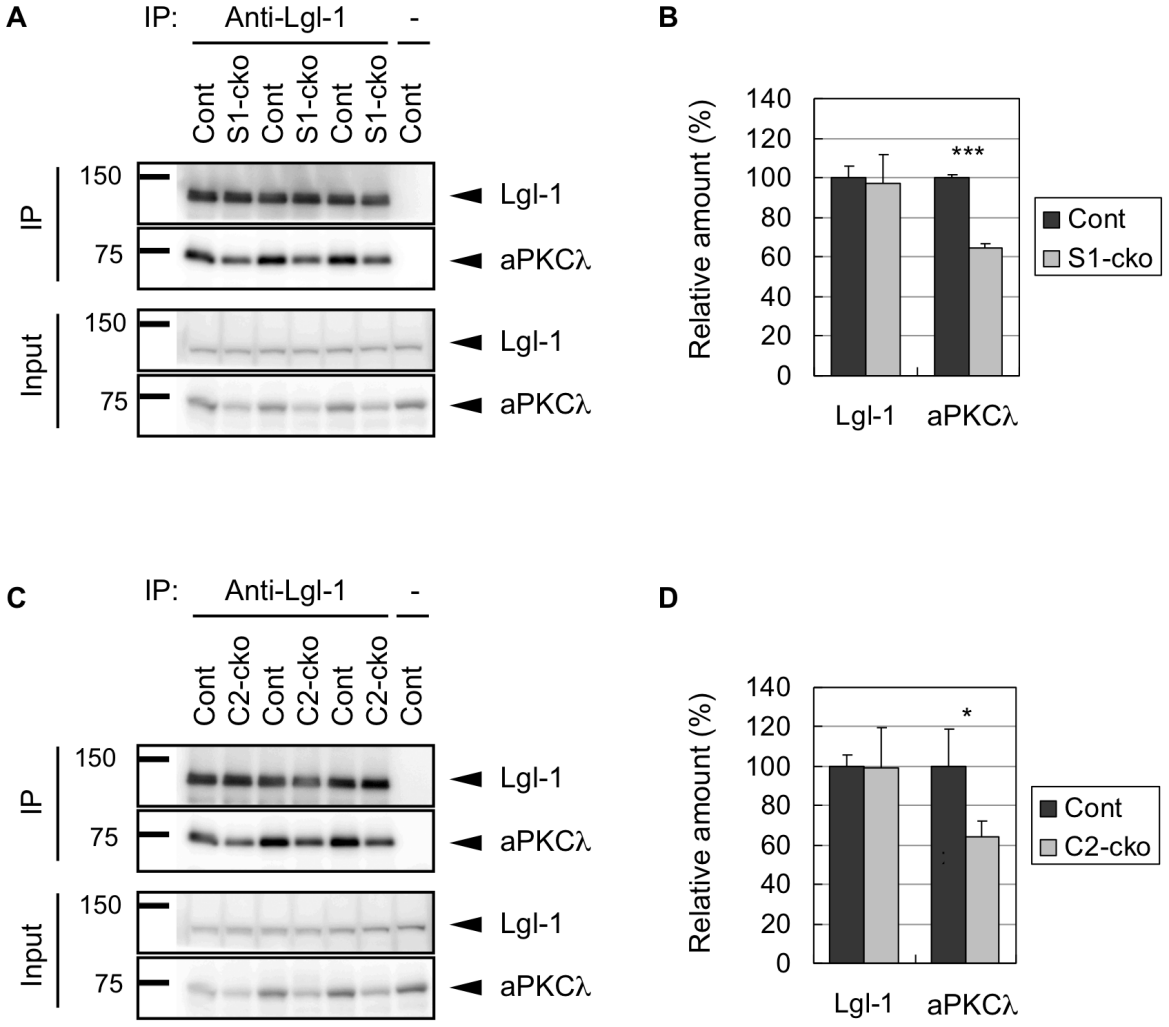
Immunoprecipitation assay using aPKCλ conditional deletion mouse brains. (A) Cerebra of 11-month-old male mice harboring aPKCλ flox/flox (Cont; n = 3) or aPKCλ flox/flox; S1-cre (aPKCλ S1-cko; n = 3) were lysed (Input) and subjected to immunoprecipitation (IP) with anti-Lgl-1 antisera. IP without antisera (-) was used as a negative control. The input and IP samples were analyzed by Western blotting using antibodies for Lgl-1 and aPKCλ. (B) Bands of IP samples in (A) were quantified and plotted. (C) Cerebra of 20-month-old male mice harboring aPKCλ flox/flox (Cont; n = 3) or aPKCλ flox/flox; C2-cre (aPKCλ C2-cko; n = 3) were subjected to IP and analyzed as in (A). (D) Bands of IP samples in (C) were quantified and plotted. Note the significant reduction of aPKCλ co-immunoprecipitated with Lgl-1 in these aPKCλ deletion mouse cerebra. Values are means ±SD (*P<0.05, ***P<0.001).

To confirm the alteration of the complex by aPKCλ deletion, we performed immunoprecipitation (IP) assay using anti-Lgl-1 antibody and found a significant reduction of aPKCλ co-immunoprecipitated with Lgl-1 in cerebra of aPKCλ S1-cko (Figure 5A, B) or C2-cko (Figure 5C, D) mice, whereas Lgl-1 in the precipitates was unchanged in these mice (Figure 5A–D). These data are compatible with those of gel filtration (Figure 4) and support the idea of disruption of the protein complex containing aPKCλ and Lgl-1 in the brain by neuronal deletion of aPKCλ.

### Neuronal deletion of aPKCλ did not induce apparent neuronal loss/degeneration in mouse brain

**Figure 6:**
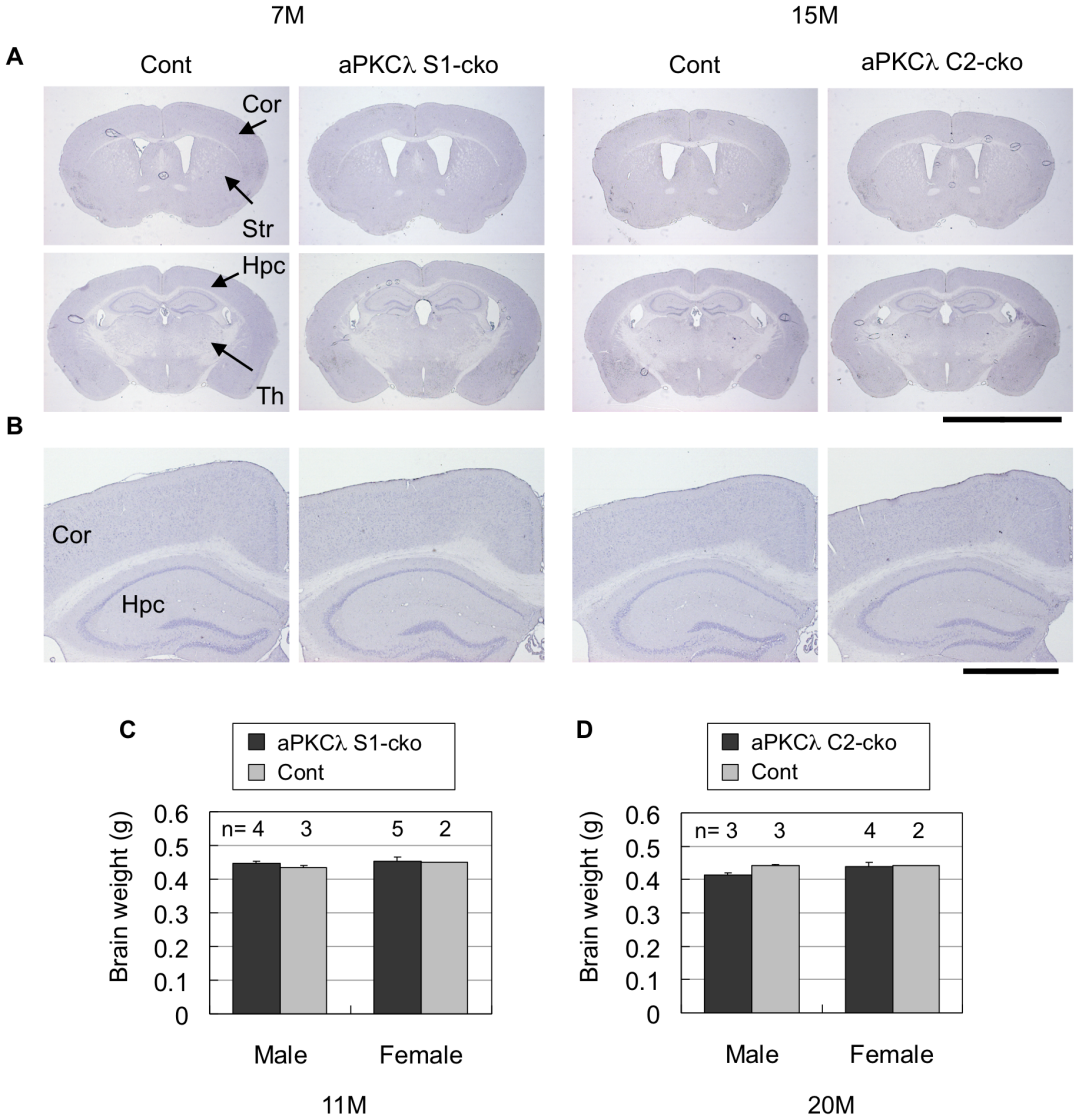
Hematoxylin staining and brain weights of aPKCλ conditional deletion mice. (A) Hematoxylin staining of coronal sections of 7-month-old aPKCλ flox/−; S1-cre (S1-cko) or flox/+ (Cont) female mice (left two panels), or 15-month-old aPKCλ flox/flox; C2-cre (C2-cko) or flox/+; C2-cre (Cont) male mice (right two panels). (B) Magnified images shown in (A). (C, D) Brain weight of 11-month-old aPKCλ flox/flox (Cont) or flox/flox; S1-cre (S1-cko) male mice (C), or 20-month-old aPKCλ flox/flox (Cont) or flox/flox; C2-cre (C2-cko) male mice (D). Numbers of mice (n) used for analysis are indicated. Values are means ± SD. Cor (cortex), Str (striatum), Hpc (hippocampus) and Th (thalamus). Bars are 5 mm (A) and 1 mm (B).

**Figure 7:**
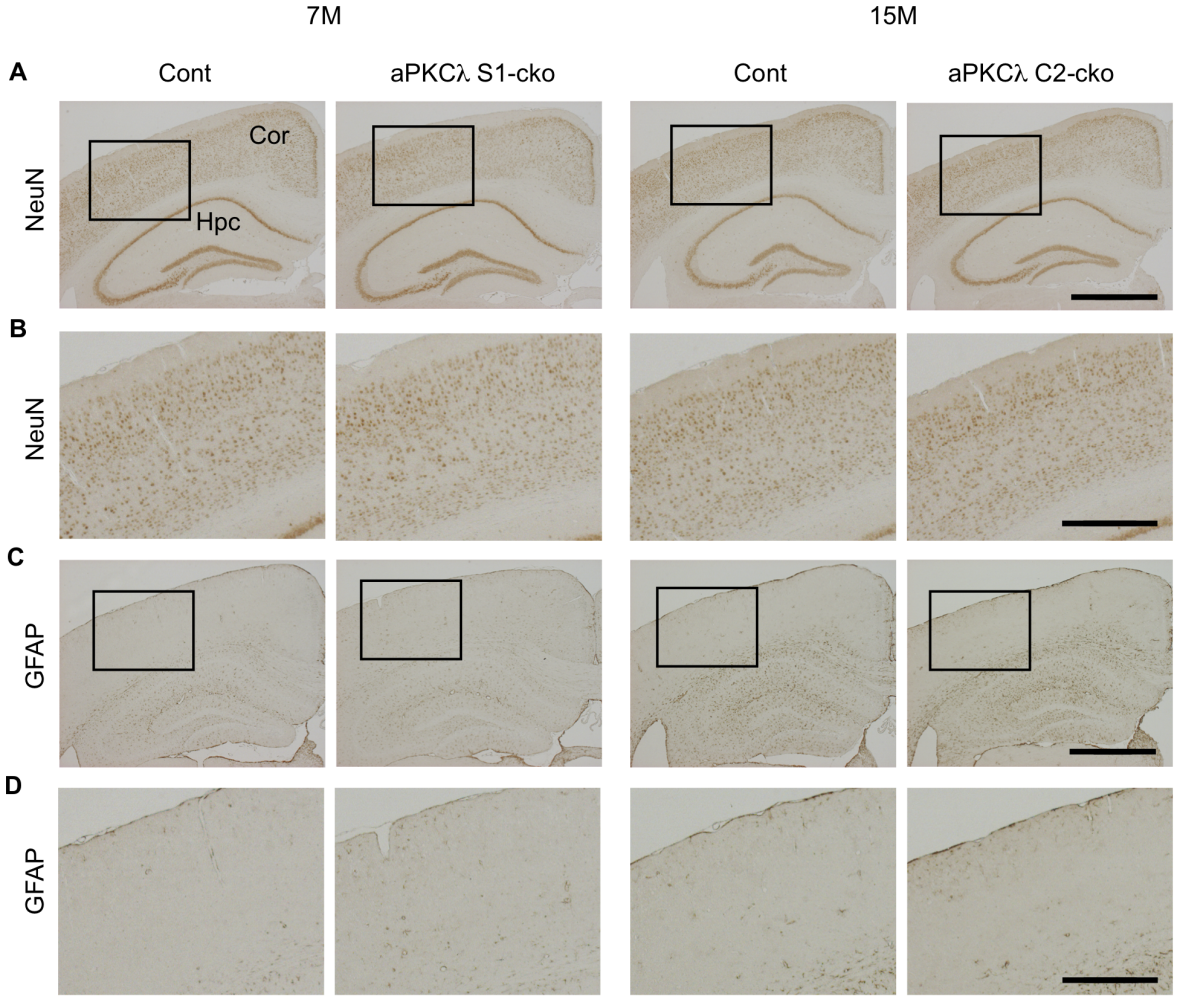
NeuN and GFAP staining of aPKCλ deletion mouse cerebrum. Immunohistochemical analysis of 7-month-old aPKCλ flox/−; S1-cre (S1-cko) or flox/+ (Cont) female mice (left two panels), or 15-month-old aPKCλ flox/flox; C2-cre (C2-cko) or flox/+; C2-cre (Cont) male mice (right two panels). (A) Staining of coronal sections with anti-NeuN, a neuronal marker. (B) Magnified images of boxed regions shown in (A). No distinct reduction of NeuN-positive cells in these aPKCλ deletion mice was found. (C) Staining of coronal sections with anti-GFAP, an astrocyte marker. (D) Magnified images of boxed regions shown in (C). No distinct induction of astrogliosis in these aPKCλ deletion mice. Cor (cortex) and Hpc (hippocampus). Bars are 1 mm (A, C) and 0.4 mm (B, D).

Although above data clearly suggest that conditional deletion of aPKCλ reduces total aPKCs and disrupts the polarity protein complex in mouse brain, neither aPKCλ S1-cko nor C2-cko mice showed any alteration in their appearance, body size or behavior (data not shown). Survival may not have been affected either (mean life spans of aPKCλ S1-cko and C2-cko female mice are 94±19 weeks (n = 6) and 98±14 weeks (n = 4), respectively). Notably, hematoxylin staining of cerebral coronal sections suggested no clear alteration in overall cell population in aPKCλ deletion mice (Figure 6A, B). In addition, total brain weights were not changed (Figure 6C, D). We also stained the sections with anti-NeuN, a neuronal marker, and found that NeuN-positive neurons seemed to be preserved in aPKCλ S1-cko and C2-cko mice (Figure 7A, B, Table S3). Furthermore, anti-GFAP staining revealed no induction of astrocytosis, an indicator of neurodegeneration (Figure 7C, D). The absence of GFAP induction was also confirmed by quantitative RT-PCR (Figure 3D, E). These data suggest that aPKCλ conditional deletion in differentiated neurons did not lead to obvious neuronal loss/degeneration in mouse brain.

### Neuronal deletion of aPKCλ may not affect neuronal orientation/distribution in mouse brain

**Figure 8:**
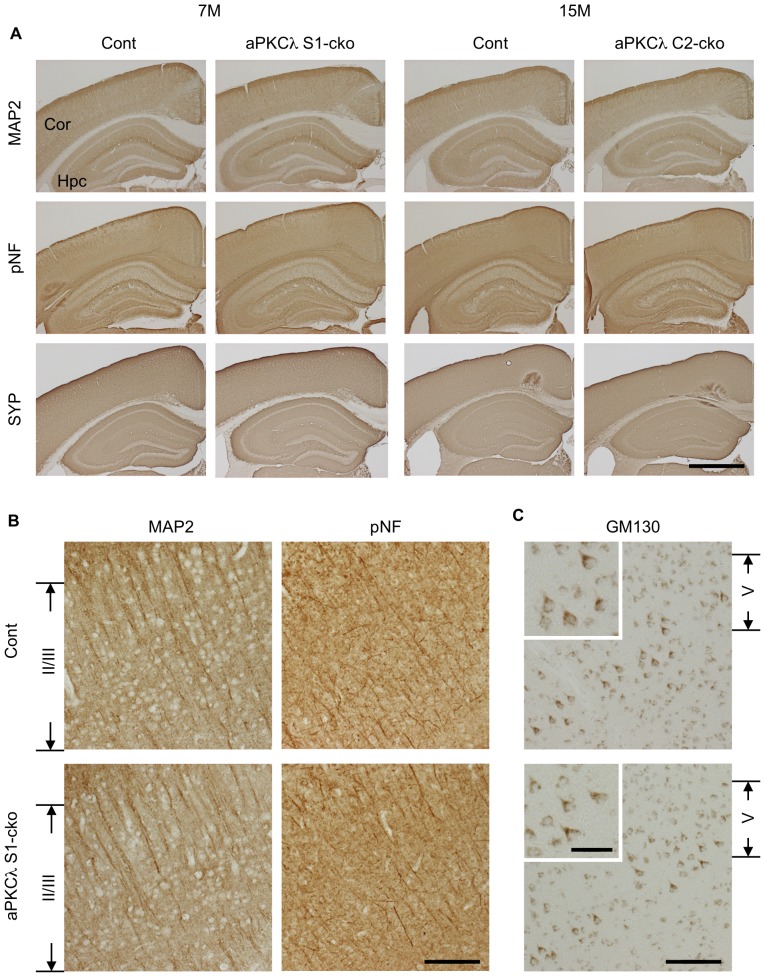
Neural marker staining of aPKCλ deletion mouse cerebrum. Immunohistochemical analysis of 7-month-old aPKCλ flox/−; S1-cre (S1-cko) or flox/+ (Cont) female mice (left two panels), or 15-month-old aPKCλ flox/flox; C2-cre (C2-cko) or flox/+; C2-cre (Cont) male mice (right two panels). (A) Staining of coronal sections with antibodies for microtubule-associated protein-2 (MAP2), phospho-neurofilament (pNF) and synaptophysin (SYP), markers for dendrites, axons and synapses (pre-synapses), respectively. (B) Enlarged images for cortical layer II/III region of 7-month-old female mice stained with anti-MAP2 or anti-pNF antibody shown in (A). (C) Staining of coronal sections of 7-month-old female mice with antibody for GM130, a Golgi marker. Images for cortical layer V region are shown, and insets are enlarged images for layer V neurons. Note no distinct alteration in neuronal marker staining and Golgi location in aPKCλ deletion mice. Cor (cortex) and Hpc (hippocampus). Bars are 1 mm (A), 100 µm (B, C) and 40 µm (insets in C).

We next examined distribution of neural structures of the aPKCλ deletion mouse brain by staining with antibodies for MAP2, phospho-neurofilaments (pNF) and synaptophysin (SYP) — markers for dendrites, axons and synapses, respectively. As shown in Figure 8A, staining patters of these proteins were not clearly altered in brains of aPKCλ S1-cko and C2-cko mice. Detailed analysis of the cortex of aPKCλ S1-cko mice suggests that distribution of dendrites and axons in layers II/III region may not be affected (Figure 8B, S2E). No distinct alteration in staining patterns of dendrites, axons and synapses was observed in aPKCλ S1-cko or C2-cko mice in later stages (Figure S2A, C, E). These data suggest that neuronal deletion of aPKCλ does not affect distribution of these neural structures in mouse brain cortex.

**Figure 9:**
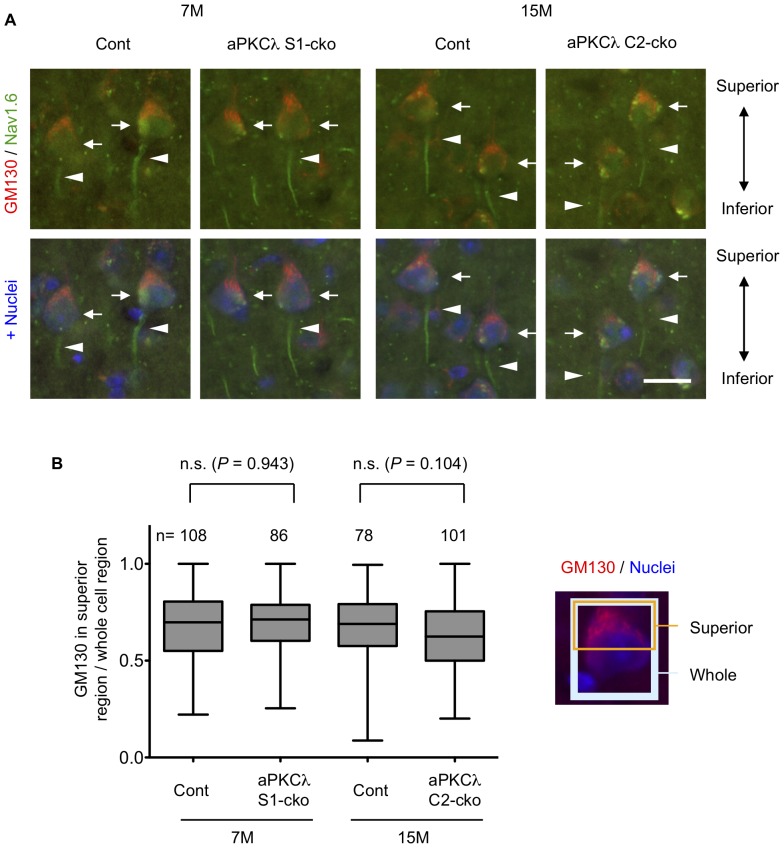
Cell orientation of cortical layer V neurons in aPKCλ deletion mice. (A) Coronal sections of 7-month-old aPKCλ flox/-; S1-cre (S1-cko) or flox/+ (Cont) female mice (left two panels), or 15-month-old aPKCλ flox/flox; C2-cre (C2-cko) or flox/+; C2-cre (Cont) male mice (right two panels) were stained with a Golgi marker GM130 (red) and an axon initial segment (AIS) marker Nav1.6 (green). Nuclei were stained with TOTO-3. Cortical layer V neurons are shown. Note that Golgi was abundant at superior part of the neurons (arrows) whereas AIS was detected in inferior region (arrowheads) in both control and aPKCλ deletion mice. (B) Immunofluorescence data in (A) were used for quantification of relative GM130 fluorescence intensities in superior region to those in whole cell region (n means number of analyzed cells). Values are means ± SD. A majority of the layer V neurons showed superior accumulation of GM130, which was not significantly affected in aPKCλ deletion mice. Bar is 20 µm (A).

**Figure 10:**
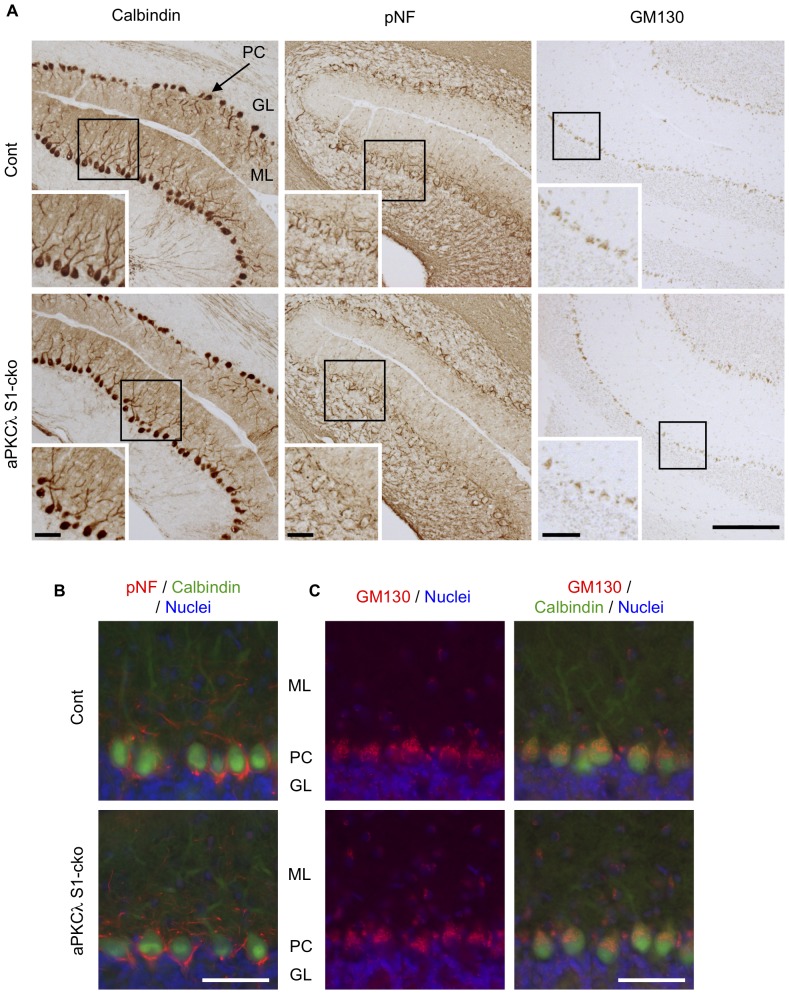
Neural marker staining of aPKCλ deletion mouse cerebellum. (A) Coronal sections of cerebellum of 7-month-old aPKCλ flox/−; S1-cre (S1-cko) or flox/+ (Cont) female mice were stained with antibodies for calbindin, phospho-neurofilament (pNF) and GM130, markers for Purkinje cells, axons and Golgi apparatus, respectively. Insets are enlarged images of boxed regions. (B, C) The sections were stained with calbindin (green) together with pNF (red; B) or GM130 (red; C). Nuclei were stained with TOTO-3. GM130 was relatively concentrated to molecular layer side in Purkinje cells, whereas pNF was highly detected in granular layer. PC (Purkinje cell), ML (molecular layer) and GL (granular layer). Bars are 200 µm (A) and 50 µm (insets of A, B, C).

We next checked cell orientation by staining with anti-GM130, a Golgi marker, and noticed that Golgi locations in layer V cortical neurons seemed to be preserved in aPKCλ S1- and C2-cko mice (Figure 8C, S2B, D, E). Detailed analysis suggested that Golgi was concentrated to the superior part of the cell body in a majority of these neurons, both in control and aPKCλ S1- or C2-cko mice (Figure 9A, B). In addition, the Nav1.6 voltage-gated sodium channel, an axon initial segment marker [50], [51], was observed at the inferior region of the neurons in both control and aPKCλ S1- or C2-cko mice (Figure 9A). Taken together, these data suggest that the orientation of layer V cortical neurons was unaffected in the aPKCλ deletion mice. We further analyzed Purkinje cells in aPKCλ S1-cko cerebellum. Calbindin and pNF staining suggest that dendritic and axonal distribution around Purkinje cells may not be affected (Figure 10A, B). In addition, the concentration of Golgi to the molecular layer side of these cells seemed to be preserved in aPKCλ S1-cko mouse (Figure 10A, C). Taken together, these data suggest that neuronal deletion of aPKCλ does not affect neuronal orientation/distribution in cortex and cerebellum.

## Discussion

In this study, we first developed mice with conditional deletion aPKCλ in brain differentiated neurons by camk2a-cre or synapsinI-cre mediated gene recombination. We found that aPKCλ is the aPKC isoform that is almost exclusively expressed in mouse brain, not aPKCζ as previously suggested [18], [49], and that neuronal deletion of aPKCλ induced reduction of the total fraction of aPKCs without inducing expression of aPKCζ and PKMζ. Biochemical analyses suggested that the aPKCλ deletion accompanied destabilization of PAR-6β and decrease in the protein complex containing aPKCλ, PAR-6β and Lgl-1. Despite the significant reductions of total aPKCs and the polarity complex, aPKCλ deletion did not induce apparent neuronal loss or degeneration in the brain, even in aged mice. In addition, staining of several markers suggested that overall neuronal orientation/distribution may be unaffected in these mice. Thus, although aPKCλ deletion in differentiated neurons disrupts the polarity complex in mouse brain, it does not induce obvious cell degeneration or neuronal disorientation, implying that aPKCλ and the polarity complex are not indispensable for neuronal survival and organized cell distribution in adult mouse brain.

Our observations stand in contrast to the critical roles of aPKC in several steps of neuronal differentiation during CNS development. One possibility is that aPKC is required only for cell polarization processes during neuronal differentiation but not for maintenance. Indeed, *in vitro* studies using cultured epithelial cells have shown that aPKCλ suppression affects cell polarity only after re-polarization [45], [52]. In addition, so far there has been no report of axonal/dendritic degeneration by suppressing aPKC or its related proteins after axonal specification in cultured hippocampal neurons [36], [37]. Alternatively, alterations are structurally too small to be detected by our tissue-based microscopic analysis. These could include dendritic spines, small protrusions forming postsynaptic structures, as aPKC and its related proteins are critical for maintenance of spine structure as well as morphogenesis in cultured hippocampal neurons [36], [37]. The essential role of aPKCλ in tissue structural maintenance is also evidenced by podocytes-specific knockout mice in which the maintenance of slit diaphragms, only detectable under an electron microscope in renal glomeruli, are disorganized, resulting in renal dysfunction [10]. Further detailed analysis is necessary for final conclusion.

One possible way to identify a clear significance of aPKCλ in differentiated neurons is examination of its role in neurological disease conditions. In epithelial cells, suppression of aPKCλ, PAR-3 or PAR-6 modulates tumorigenesis of several tissues *in vivo* [53]–[59]. Morphological abnormalities of dendritic spines are reported in a variety of neurological diseases such as Fragile-X mental retardation syndrome and Alzheimer's neurodegenerative disease [60], [61], and notably functional interaction of FMR1, a causative gene of Fragile-X syndrome, with Lgl is reported in *Drosophila* [62]. Tau-mediated neuropathology is also possible because PAR-1, a downstream target of aPKC [63], [64], is shown to be involved in hyperphosphorylation of tau which links to Alzheimer's disease [65]. Axon regeneration after CNS injury may also be interesting for analysis because roles of aPKCλ in axonal elongation and guidance have been reported [66]–[68].

Very recently, Ren et al. have shown that knockdown of aPKCλ in hippocampal neurons suppresses expression of long-term potentiation (LTP), and in this case aPKCλ cooperates with p62 for phosphorylation of AMPA receptors to mediate its synaptic incorporation [69]. Because p62 is another protein that binds to the PB1 domain of aPKCλ in addition to PAR-6 [46], [47], it seems that this aPKCλ function is different from that in the cell polarity complex with PAR-6. An aPKC inhibitory peptide, aPKC pseudosubstrate (PS) peptide, suppresses PKMζ and induces LTP suppression and memory perturbation [48]. However, two groups have recently reported that knockout of aPKCζ/PKMζ does not affect LTP and learning/memory in mice whereas aPKC-PS peptide is still effective in these mice [49], [70]. In addition, aPKC-PS peptide is also shown to suppress aPKCλ at physiological concentrations [49], [69]. Thus, it is likely that aPKCλ is also the physiological target of aPKC-PS peptide and involved in memory function by regulating LTP in mouse brain. Our mutant mice may be useful in examining polarity-independent functions of aPKCλ, which would identify novel mechanisms underlying maintenance of long-term memory *in vivo*.

## Materials and Methods

### Mice

The mouse experiments were approved by the animal experiment committee at RIKEN Brain Science Institute. Mice were maintained and bred in accordance with RIKEN guidelines. The generation of aPKCλ flox mice maintained on a C57BL6 (B6) background was described previously [18]. The transgenic mice for camk2a-cre (C2-cre) harboring a cre transgene under the camk2a promoter (B6.Cg-Tg (Syn1-cre) 671Jxm/J) [41] and mice for synapsinI-cre (S1-cre) harboring a cre transgene under the synapsinI promoter (B6,Cg-Tg (Camk2a-cre) T29-qStl/J) [42] were obtained from the Jackson Laboratory (Bar Harbor, ME). RNZ (ROSA26-loxP-STOP-loxP-nlsLacZ) knock-in (KI) mice that express LacZ under cre-mediated recombination [43] were generously provided by Dr. Itohara (RIKEN BSI). All mice were maintained on a B6 background. For generation of C2-cre-medicated aPKCλ conditional deletion (aPKCλ C2-cko) mice, we crossed aPKCλ flox/flox mice with aPKCλ flox/+; C2-cre mice. For generation of S1-cre-medicated aPKCλ conditional deletion (aPKCλ S1-cko) mice, we crossed aPKCλ flox/flox mice with aPKCλ flox(-)/+; C2-cre. The (-) indicates a deleted allele of aPKCλ detected in some mice when crossed with S1-cre during generation, possibly due to germline recombination [44]. As a consequence, mice with a deleted aPKCλ allele (-) instead of the flox allele were occasionally obtained in the generation of aPKCλ S1-cko mice. The sequences of primers used for genotyping are listed in Table S4.

### Antibodies

Rabbit polyclonal antibodies for PAR-6β (BC31AP) and Lgl-1 (C-2AP) were described previously [6]. Rabbit polyclonal antibody for Nav1.6 was generously provided by Dr. Ogiwara and Dr. Yamakawa (RIKEN BSI) [50], [51]. Antibodies for aPKCλ (610175) and GM130 (610822) were from BD (Transduction); and synaptophysin (SYP, MAB5258), Calbindin D-28K (AB1778) and NeuN (MAB377) were from MILLIPORE (Chemicon). The following antibodies were also used: β-actin (A5441, Sigma-Aldrich), aPKCλ/ζ (C-20) (sc-216, Santa Cruz), GFAP (Z0334, DAKO), LacZ (200-4136, Rockland), MAP2 (M4403, Sigma-Aldrich), p62 (PM045, MBL), PAR-3 (07-330, Upstate) and phospho-neurofilament (pNF) (SMI 31, Covance (Sternberger Monoclonals Inc)).

### Histological analysis

Mice were perfused with 4% paraformalxehyde (PFA)/phosphate-buffered saline (PBS), cryoprotected with 20% sucrose/PBS and processed for cryosectioning (10 µm or 20 µm). Hematoxylin staining was performed using Mayer's Hematoxylin. Immunohistochemistry and immunofluorescence microscopy were performed as described previously [71], [72], and images were obtained by a CCD camera-equipped Olympus microscope (AX80) or Keyence microscope (BZ-9000). Quantitative analyses (counting of anti-NeuN-positive cells and measurement of anti-GM130 fluorescence intensities) were performed using ImageJ software [73]. For LacZ staining, fixed whole brains by perfusion were cut into 2-mm sections using brain matrix, and further fixed in 4% PFA/PBS for 2 hr at 4°C. After rinsing with 100 mM NH_4_Cl/PBS and detergent solution (2 mM MgCl_2_, 0.01% deoxycholate, 0.02% NP-40 in PBS), sections were incubated in X-gal solution (1 mg/ml X-gal, 5 mM pottasium ferrocyanide, 5 mM pottasium ferricyanide in detergent solution) overnight at 37°C. Images were obtained using a digital camera-equipped Leica stereo microscope (MZFLIII).

### Quantitative reverse transcription (RT)-PCR

Preparations of total RNA, reverse transcription and cDNA synthesis from mouse tissue were performed as described previously [71]. Primers for quantitative real-time PCR were designed based on Primer Express software (Applied Biosystems). Real-time PCR was performed by Roche FastStart Universal SYBR Green Master (ROX) using LightCycler 480 (Roche) according to the manufacturer's protocol. All values obtained were normalized with respect to levels of GAPDH mRNA. Primers used for RT-PCR are listed in Table S5. Plasmid DNA for mouse aPKCλ or mouse aPKCζ in SRD vector was used to check specificities of primers for detection of aPKCλ and aPKCζ and to compare the amount of full-length aPKCζ with that of PKMζ in mouse brain.

### Gel filtration, immunoprecipitation and Western blotting

For gel filtration, isolated brain cortexes were homogenized in phosphate-buffered saline (PBS) containing 0.1% triton X-100 and complete protease inhibitor on ice. After centrifugation at 14 krpm for 30 min and filtration with 0.45 µm filter, the lysates containing 250 µg of protein were separated by gel filtration (superose 6) using a SMART system (GE Pharmacia) at a speed of 40 µl/min. Total 40 fractions (40 µl/tube) were collected from 18 min after the sample loading. For immunoprecipitation, brain cerebra were homogenized in lysis buffer containing 20 mM Hepes at pH 7.2, 150 mM NaCl, 0.5% triton X-100, 10% glycerol and complete protease inhibitor. After centrifugation at 14 krpm for 30 min, the lysates containing 2 mg of protein were co-incubated with anti-Lgl-1 antisera (C-2) conjugated with protein A sepharose. After washing with the lysis buffer three times, the immunoprecipitates were eluted with SDS sample buffer. SDS-PAGE and Western blotting were performed as described previously [71]. Chemiluminescent signals were obtained and quantified using ImageQuant LAS-4000 (GE).

### Statistical analysis

For comparison between two sample groups, data were first analyzed by F-test. For *P*<0.05, the data were analyzed by unpaired Student's t-test (two-tailed); otherwise data were analyzed by Welch's t-test (two-tailed). We considered the difference between comparisons to be significant when *P*<0.05 for all statistical analyses.

## Supporting Information

Figure S1 **LacZ staining of RNZ mice harboring synapsinI-cre or camk2a-cre.** RNZ mice harboring synapsinI-cre (S1-cre) or camk2a-cre (C2-cre) were subjected to LacZ staining using X-gal as a substrate to detect cre-mediated DNA recombination. (A) Wide distribution of LacZ-positive cells in brain of 18 week-old S1-cre; RNZ female mouse. (B) Magnified images of cortex, hippocampus and cerebellum shown in (A). (C) Forebrain-specific distribution of LacZ-positive cells in brain of 8 week-old C2-cre; RNZ female mouse. Age-matched RNZ female mouse (without cre transgene) was used as a negative control. (D) Magnified images of cortex, striatum and hippocampus shown in (C). Cor (cortex), Str (striatum), Hpc (hippocampus), Th (thalamus), Cbl (cerebellum), BS (brain stem), and DG (dentate gyrus). Bars are 5 mm (A, C) and 1 mm (B, D).
(TIFF)

Figure S2 **Neural marker staining of cerebrum of aged aPKCλ deletion mice.** Immunohistochemical analysis of 18-month-old aPKCλ flox/−; S1-cre (S1-cko) or flox/+ (Cont) male mice (A, B), or 26-month-old aPKCλ flox/flox; C2-cre (C2-cko) or flox/+ (Cont) female mice (C, D). (A, C) Staining of coronal sections with antibodies for microtubule-associated protein-2 (MAP2), phospho-neurofilament (pNF) and synaptophysin (SYP), markers for dendrites, axons and synapses (pre-synapses), respectively. Images for cortical layer II/III region are shown. (B, D) Staining of coronal sections with antibody for GM130, a Golgi marker. Images for cortical layer V region are shown (insets are enlarged images of layer V neurons). Note no distinct alteration in neuronal marker staining and Golgi localization in aPKCλ deletion mouse. (E) Cortical areas shown in (A, C) containing layers II/III and in (B, D) containing layer V. Cor (cortex) and Hpc (hippocampus). Bars are 100 µm (A–D) and 40 µm (insets in B, D).
(TIFF)

Table S1 **Born ratio of aPKCλ C2-cko mice.**
(PDF)

Table S2 **Born ratio of aPKCλ S1-cko mice.** *The (-) means deleted allele of aPKCλ detected in some mice when crossed with S1-cre possibly due to its recombination in germline. ^†^Mice with aPKCλ deleted allele (-) instead of flox allele were occasionally obtained during generation.
(PDF)

Table S3 **Quantification of anti-NeuN stained cells in brain cortex.** *Coronal sections of indicated control or aPKCλ deletion mice were stained with anti-NeuN. The NeuN-positive cells in all layers of cortex (60 µm in width) in left and right hemisphere were quantified. Mean cell number and ratio to control for each pair were also indicated.
(PDF)

Table S4 **List of primers used for genotyping.**
(PDF)

Table S5 **List of primers used for quantitative RT-PCR.**
(PDF)
