# Supplementary figures and images for: Loss of aPKCλ in Differentiated Neurons Disrupts the Polarity Complex but Does Not Induce Obvious Neuronal Loss or Disorientation in Mouse Brains
Source: PLoS One. 2013 Dec 31;8(12):e84036. doi: 10.1371/journal.pone.0084036 (PMC3877147; doi:10.1371/journal.pone.0084036)

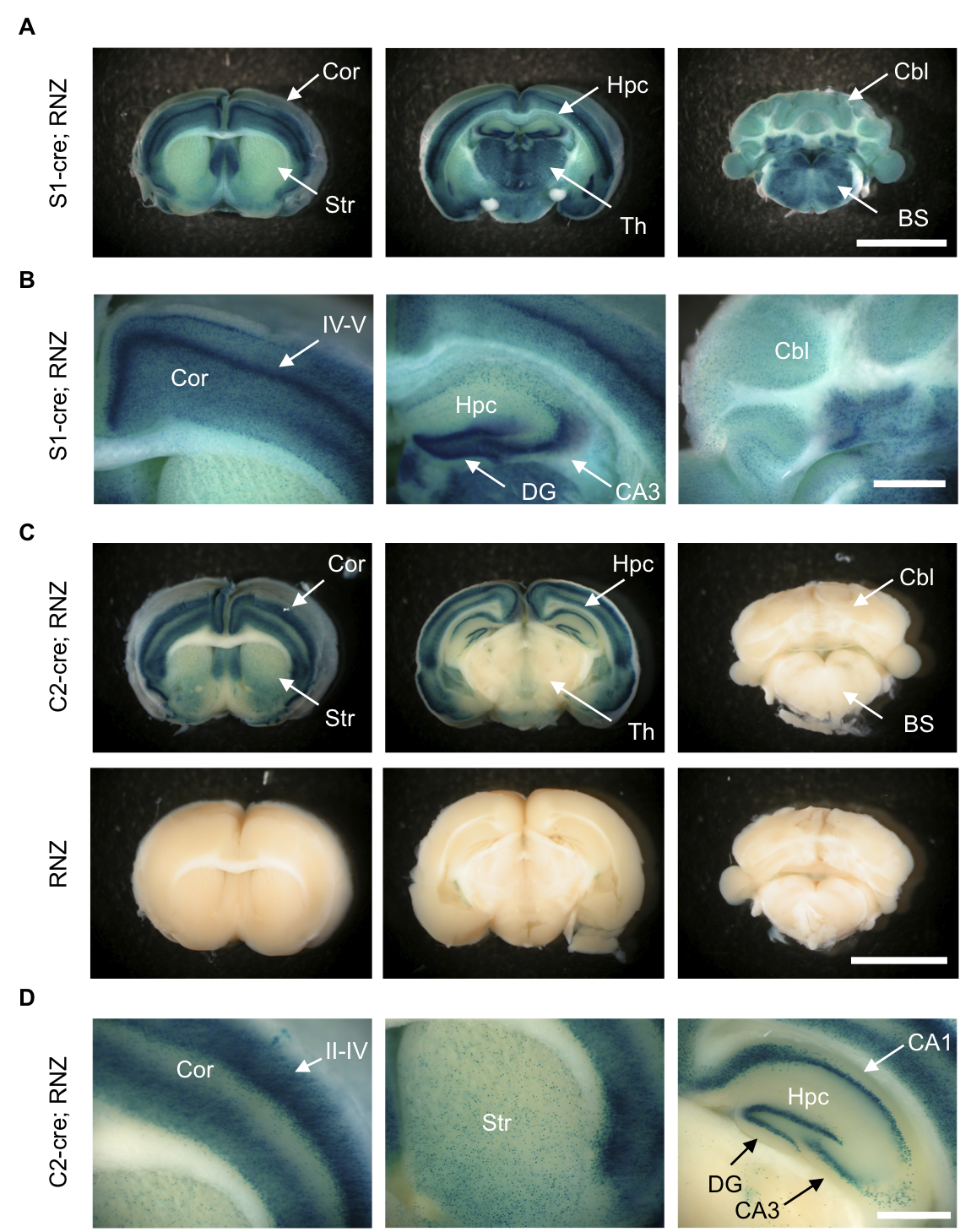

Supplement: Figure S1 — LacZ staining of RNZ mice harboring synapsinI-cre or camk2a-cre. RNZ mice harboring synapsinI-cre (S1-cre) or camk2a-cre (C2-cre) were subjected to LacZ staining using X-gal as a substrate to detect cre-mediated DNA recombination. (A) Wide distribution of LacZ-positive cells in brain of 18 week-old S1-cre; RNZ female mouse. (B) Magnified images of cortex, hippocampus and cerebellum shown in (A). (C) Forebrain-specific distribution of LacZ-positive cells in brain of 8 week-old C2-cre; RNZ female mouse. Age-matched RNZ female mouse (without cre transgene) was used as a negative control. (D) Magnified images of cortex, striatum and hippocampus shown in (C). Cor (cortex), Str (striatum), Hpc (hippocampus), Th (thalamus), Cbl (cerebellum), BS (brain stem), and DG (dentate gyrus). Bars are 5 mm (A, C) and 1 mm (B, D). (TIFF) [file pone.0084036.s001.tiff]

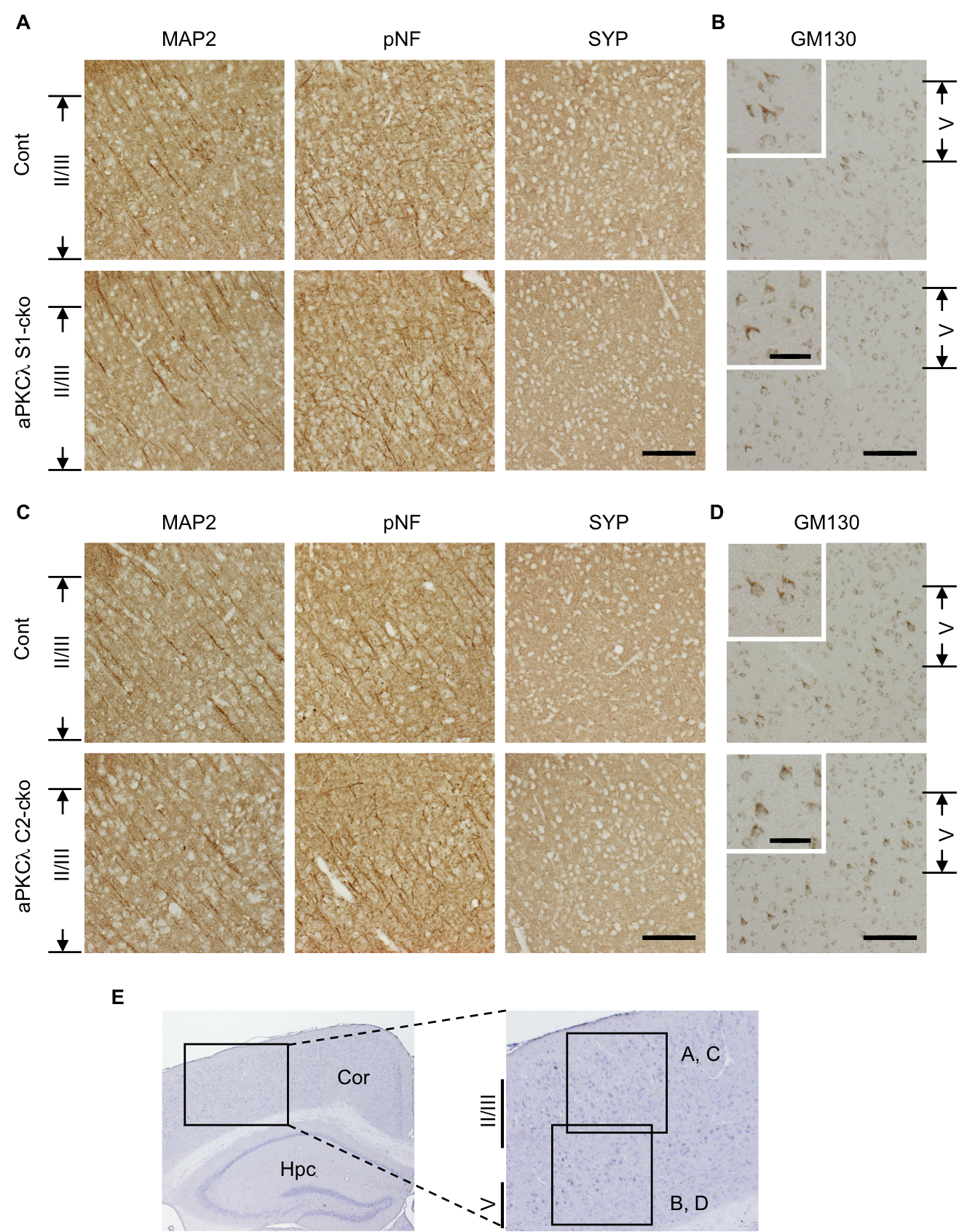

Supplement: Figure S2 — Neural marker staining of cerebrum of aged aPKCλ deletion mice. Immunohistochemical analysis of 18-month-old aPKCλ flox/−; S1-cre (S1-cko) or flox/+ (Cont) male mice (A, B), or 26-month-old aPKCλ flox/flox; C2-cre (C2-cko) or flox/+ (Cont) female mice (C, D). (A, C) Staining of coronal sections with antibodies for microtubule-associated protein-2 (MAP2), phospho-neurofilament (pNF) and synaptophysin (SYP), markers for dendrites, axons and synapses (pre-synapses), respectively. Images for cortical layer II/III region are shown. (B, D) Staining of coronal sections with antibody for GM130, a Golgi marker. Images for cortical layer V region are shown (insets are enlarged images of layer V neurons). Note no distinct alteration in neuronal marker staining and Golgi localization in aPKCλ deletion mouse. (E) Cortical areas shown in (A, C) containing layers II/III and in (B, D) containing layer V. Cor (cortex) and Hpc (hippocampus). Bars are 100 µm (A–D) and 40 µm (insets in B, D). (TIFF) [file pone.0084036.s002.tiff]
