# Supplementary material for: Loss of aPKCλ in Differentiated Neurons Disrupts the Polarity Complex but Does Not Induce Obvious Neuronal Loss or Disorientation in Mouse Brains
Source: PLoS One. 2013 Dec 31;8(12):e84036. doi: 10.1371/journal.pone.0084036 (PMC3877147; doi:10.1371/journal.pone.0084036)
Supplement: Table S1 — Born ratio of aPKCλ C2-cko mice. (PDF) [file pone.0084036.s003.pdf]

Table S1. Born ratio of aPKCλ C2-cko mice.

aPKCλ flox/flox x aPKCλ flox/+; C2-cre							
	All		Male		Female		Expected ratio
	No	Ratio	No	Ratio	No	Ratio	
flox/flox; C2-cre	24	24.5	11	21.6	13	27.7	25
flox/+; C2-cre	24	24.5	15	29.4	9	19.1	25
flox/flox	24	24.5	13	25.5	11	23.4	25
flox/+	26	26.5	12	23.5	14	29.8	25
