# Supplementary material for: Loss of aPKCλ in Differentiated Neurons Disrupts the Polarity Complex but Does Not Induce Obvious Neuronal Loss or Disorientation in Mouse Brains
Source: PLoS One. 2013 Dec 31;8(12):e84036. doi: 10.1371/journal.pone.0084036 (PMC3877147; doi:10.1371/journal.pone.0084036)
Supplement: Table S2 — Born ratio of aPKCλ S1-cko mice. *The (-) means deleted allele of aPKCλ detected in some mice when crossed with S1-cre possibly due to its recombination in germline. †Mice with aPKCλ deleted allele (-) instead of flox allele were occasionally obtained during generation. (PDF) [file pone.0084036.s004.pdf]

**Table S2. Born ratio of aPKCλ S1-cko mice.**

| aPKCλ flox/flox x aPKCλ flox/+(-); S1-cre* | | | | | | | |
|---|---|---|---|---|---|---|---|
| | All | | Male | | Female | | Expected ratio |
| | No | Ratio | No | Ratio | No | Ratio | |
| flox/flox; S1-cre (or flox/-; S1-cre)† | 30 | 35.7 | 11 | 32.3 | 19 | 38.0 | 25 |
| flox/+; S1-cre (or +/-; S1-cre)† | 19 | 22.6 | 8 | 23.5 | 11 | 22.0 | 25 |
| flox/flox (or flox/-)† | 19 | 22.6 | 8 | 23.5 | 11 | 22.0 | 25 |
| flox/+ | 16 | 19.0 | 7 | 20.5 | 9 | 18.0 | 25 |

*The (-) means deleted allele of aPKCλ detected in some mice when crossed with S1-cre possibly due to its recombination in germline. †Mice with aPKCλ deleted allele (-) instead of flox allele were occasionally obtained during generation.
