# Supplementary material for: Loss of aPKCλ in Differentiated Neurons Disrupts the Polarity Complex but Does Not Induce Obvious Neuronal Loss or Disorientation in Mouse Brains
Source: PLoS One. 2013 Dec 31;8(12):e84036. doi: 10.1371/journal.pone.0084036 (PMC3877147; doi:10.1371/journal.pone.0084036)
Supplement: Table S3 — Quantification of anti-NeuN stained cells in brain cortex. *Coronal sections of indicated control or aPKCλ deletion mice were stained with anti-NeuN. The NeuN-positive cells in all layers of cortex (60 µm in width) in left and right hemisphere were quantified. Mean cell number and ratio to control for each pair were also indicated. (PDF) [file pone.0084036.s005.pdf]

Table S3. Quantification of anti-NeuN stained cells in brain cortex.

	Genotype	Age	NeuN-positive cells*			Ratio to control
			Left	Right	Mean	
Cont	aPKCλ flox/+	7 months	697	713	705	
S1-cko	aPKCλ flox/-; S1-cre	7 months	703	703	703	0.997
Cont	aPKCλ flox/+	18 months	735	699	717	
S1-cko	aPKCλ flox/flox; S1-cre	18 months	658	717	687.5	0.959
Cont	aPKCλ flox/+; C2-cre	15 months	766	666	716	
C2-cko	aPKCλ flox/flox; C2-cre	15 months	736	643	689.5	0.963
Cont	aPKCλ flox/+	26 months	738	737	737.5	
C2-cko	aPKCλ flox/flox; C2-cre	26 months	735	749	742	1.006

*Coronal sections of indicated control or aPKCλ deletion mice were stained with anti-NeuN. The NeuN-positive cells in all layers of cortex (600 μm in width) in left and right hemisphere were quantified. Mean cell number and ratio to control for each pair were also indicated.
