# Supplementary material for: Loss of aPKCλ in Differentiated Neurons Disrupts the Polarity Complex but Does Not Induce Obvious Neuronal Loss or Disorientation in Mouse Brains
Source: PLoS One. 2013 Dec 31;8(12):e84036. doi: 10.1371/journal.pone.0084036 (PMC3877147; doi:10.1371/journal.pone.0084036)
Supplement: Table S4 — List of primers used for genotyping. (PDF) [file pone.0084036.s006.pdf]

Table S4. List of primers used for genotyping.

Genotype	Primer forward		Primer reverse	
aPKCλ WT, Flox	flox-L62	CATGCAGTGTACTGGCATAGCCACC	flox-L64	AGAGGCAGCCAAAGCCCTGCTCTCC
aPKCλ Del	del-L57	GTCGTCTGTGTGCCTGCCGTAACTG	del-L63	CAAAGCCCTGCTCTCCTAGAGCCTG
Cre	cre-fw	AGATGTTCGCGATTATC	cre-rv	AGCTACACCAGAGACGG
RNZ WT	R523	GGAGCGGGAGAAATGGATATG	R26F2	AAAGTCGCTCTGAGTTGTTAT
RNZ KI	R1295	GCGAAGAGTTTGTCCTCAACC	R26F2	AAAGTCGCTCTGAGTTGTTAT
