# Supplementary material for: Loss of aPKCλ in Differentiated Neurons Disrupts the Polarity Complex but Does Not Induce Obvious Neuronal Loss or Disorientation in Mouse Brains
Source: PLoS One. 2013 Dec 31;8(12):e84036. doi: 10.1371/journal.pone.0084036 (PMC3877147; doi:10.1371/journal.pone.0084036)
Supplement: Table S5 — List of primers used for quantitative RT-PCR. (PDF) [file pone.0084036.s007.pdf]

Table S5. List of primers used for quantitative RT-PCR.

	Forward	Reverse
aPKCλ	CGGTGGAGAAAGCTGTATTGTG	TGGGCGCGCCTATTGA
aPKCζRD	GCATGCCTTGTCCTGGAGAA	CGGTACAGCTTCCTCCATCTTC
aPKCζKD	CTGGGTGTCCTTATGTTTGAGATG	GTCAGGGTTGTCCGTGATGAT
GAPDH	TGTGTCCGTCGTGGATCTGA	CCTGCTTCACCACCTTCTTGA
GFAP	CCAGCTTCGAGCCAAGGA	GAAGCTCCGCCTGGTAGACA
Lgl-1	GGCCAGGGCTTTTACTTGATT	TGTGATGTTGCGAGCACTCA
PAR-3	TGCGCGTGTCATTTGGTT	CTCGCGTTGGGACAGTTGT
PAR-6α	CAACAGCCACAACCTCATCGT	GCCCCCCGTACCACATTAT
PAR-6β	AACAGCCGCAACCTCATCA	TTGCGCACCACGTTGTTC
